# Unveiling Another Missing Piece in EBV-Driven Lymphomagenesis: EBV-Encoded MicroRNAs Expression in EBER-Negative Burkitt Lymphoma Cases

**DOI:** 10.3389/fmicb.2017.00229

**Published:** 2017-03-01

**Authors:** Lucia Mundo, Maria R. Ambrosio, Matteo Picciolini, Giuseppe Lo Bello, Sara Gazaneo, Leonardo Del Porro, Stefano Lazzi, Mohsen Navari, Noel Onyango, Massimo Granai, Cristiana Bellan, Giulia De Falco, Davide Gibellini, Pier P. Piccaluga, Lorenzo Leoncini

**Affiliations:** ^1^Section of Pathology, Department of Medical Biotechnology, University of SienaSiena, Italy; ^2^Diatech PharmacogeneticsJesi, Italy; ^3^Department of Experimental, Diagnostic, and Experimental Medicine, S. Orsola-Malpighi Hospital, Bologna University School of MedicineBologna, Italy; ^4^Department of Clinical Medicine and Therapeutics, University of NairobiNairobi, Kenya; ^5^School of Biological and Chemical Sciences, Queen Mary University of LondonLondon, UK; ^6^Virology Unit, Department of Diagnostic and Public Health, University of VeronaVerona, Italy; ^7^Euro-Mediterranean Institute of Science and TechnologyPalermo, Italy

**Keywords:** Epstein–Barr virus, Burkitt lymphoma, hit-and-run, microRNA expression profiling, vaccines

## Abstract

Epstein–Barr virus (EBV) is a gammaherpesvirus linked to a number of lymphoid and epithelial malignancies, including Burkitt lymphoma (BL) in which its frequency ranges from 30% in sporadic cases to 100% in the endemic ones. The possible contribution of EBV to BL pathogenesis is largely unknown. It has been suggested that EBV may be associated with all of the cases, including those diagnosed as EBV negative by a mechanism of *hit-and-run*. Early during oncogenesis, viral genes are essential for initiating disease. Progressively, viral genome is lost to escape the immune system and host mutations accumulate in proto-oncogenic cell. The main problem with the *hit-and-run* hypothesis is the lack of evidence in primary tumors. The routine methods applied to detect the virus [i.e., immunohistochemistry and EBV-encoded RNAs (EBER) *in situ* hybridization (ISH)] have a low specificity and accuracy. The aim of this study was to identify the most suitable method to detect EBV infection in pathology samples by applying conventional and non-conventional methods (i.e., EBV-microRNAs detection and EBV viral load measurement). We investigated a total of 10 cases and we found that all the samples (*n* = 6) diagnosed as EBV negative by immunohistochemistry and EBER-ISH demonstrated the presence of EBV-microRNAs and EBV genome. This points at the possibility that EBV might have contributed to lymphomagenesis in all our patients, and propose microRNAs detection as the most specific and sensitive tool to recognize EBV vestiges. It is worth noting that our data would have considerable implications for EBV-related diseases control. By using anti-EBV vaccines, one could potentially prevent also some cancers less suspected of a viral origin because of viral genome loss.

## Introduction

Epstein–Barr virus (EBV) is a gammaherpesvirus that persistently infects over 90% of adults, usually without consequence (Queen et al., 2013). However, EBV is linked to a diverse array of lymphoid and epithelial malignancies, including Burkitt lymphoma (BL) (Queen et al., 2013). The virus is detected in 30% of sporadic BL (sBL), 50% of immunodeficiency associated BL (ID-BL) and virtually all endemic BL (eBL) (Leoncini et al., 2008). The possible contribution of EBV to BL pathogenesis is largely unknown and it is unclear how directly infection and disease are linked (Rochford et al., 2005). In fact, viral genes seem mostly to have triggering or accessory roles in disease, and are likely to be essential for cancer-cell survival only in the early phase of the neoplastic transformation in non-immunocompromised carriers (Vereide and Sugden, 2010). Moreover, the role of EBV is further confounded by the less than total association of the virus with histologically similar tumors. This may be explained by the *hit-and-run* hypothesis for viral-induced lymphomagenesis which proposes that after eliciting a heritable change in the gene-expression pattern of the host cell, the genome of tumor viruses may be completely lost (Ambinder, 2000; Minarovits et al., 2011). Following, cancers accumulate vast numbers of host mutations which become the main drivers of oncogenesis, promoting autonomous growth (Minarovits et al., 2011). Thus, it seems inevitable that a cancer, with time, will evolve to be independent from viral gene functions, allowing viral genome loss. This results in an inverse correlation between the number of viral genes expressed in these tumor cells and their associated cellular mutations (Rochford et al., 2005) as it has been recently demonstrated in cell lines and tumor samples (Abate et al., 2015). The main problem with the *hit-and-run* hypothesis has been lack of evidence in primary tumors; in addition, the studies present in the literature on the role of EBV in tumorigenesis have analyzed mainly EBV-positive cancers. Focusing on virus-positive cancers provide little information about genome loss, and the difficulty of analyzing spontaneous cancers, where the molecular changes driving transformation are almost always unknown, makes firm functional conclusions hard to draw (Stevenson et al., 2010).

To assess the presence of the virus in a specific sample, different approaches can be used, most of which are characterized by a high sensitivity. To date, the most employed methods for diagnostic purposes are immunohistochemistry and *in situ* hybridization (ISH) for EBV-encoded RNAs (EBER). However, they have a low specificity, and the accuracy of such assays has been recently called into question by molecular studies that showed the presence of the virus in samples previously diagnosed as EBER negative (Gallagher et al., 2003; Qi et al., 2013). More recently, microRNA (miRNA) expression has been shown to be a quite sensitive and specific tool to characterize normal and neoplastic cells, even for pathogens detection (Ma et al., 2016). By miRNA profiling we recently observed the expression of EBV-encoded miRNAs in one EBER-negative BL case (Abate et al., 2015; Piccaluga et al., 2016). Based on that, the aim of the present study was to identify the most suitable method to detect EBV infection in pathology samples by comparing different conventional (immunohistochemistry and EBER-ISH) and non-conventional (EBV-microRNAs detection and EBV viral load measurement) methods. We looked for EBV infection in 10 typical BL cases. Immunohistochemistry and EBER-ISH failed to identify the virus in six samples, whereas microRNAs expression profiling, quantitative reverse transcription PCR and viral load measurement identified a previous EBV exposure in all the specimens, also in those diagnosed as EBV negative by conventional tools. Our findings shed new light on the pathogenesis of EBV-related tumors, highlighting the role of the virus also in “EBV-negative” cases and proposing EBV-miRNAs searching as the most sensitive approach to identify also EBV vestiges. Because of the limitation of using EBV-miRNAs detection routinely, there is a lack of data to determine the true burden of EBV-associated cancers worldwide, and the data available is likely to under represent the epidemiology of EBV infection. Assessing that EBV might be responsible of a larger number of cancers than previously known may open the way for the opportunity of a large scale prevention tactic.

## Materials and Methods

### Patients

The cases cohort was represented by 10 formalin-fixed and paraffin-embedded (FFPE) samples, retrieved by the Archives of Siena University Hospital, and characterized by clinic, morphology, immunohenotype and cytogenetic consistent with the World Health Organization diagnosis of BL. The mean age at diagnosis was 14.2 years (range: 3–41 years) with a male to females ratio of 5:5. The sites of involvement were: small intestine (*n* = 4), lymph node (*n* = 2), head and neck (*n* = 1), ovary (*n* = 1), stomach (*n* = 1), uterus (*n* = 1). None of the patient had underlying immunosuppression. All individuals were positive for IgG antibodies against viral capsid antigen (VCA) and EBV nuclear antigen (EBNA), whereas they were negative for IgM antibodies against VCA and early antigen (EA).

### Immunohistochemistry and EBER-ISH

Immunohistochemical stainings were performed on all cases by an automated staining system (Ventana BenchMark ULTRA, Roche diagnostic, Monza-Italy) on FFPE 4 μm-thick sections with appropriate positive and negative controls included in each staining run. No epitope retrieval was exploited. UltraView Universal Detection Kit (Ventana) using HRP multimer and DAB (as chromogen) was employed. Each case was scored as positive or negative for EBNA-1 (Santa Cruz, Heidelberg, Germany). ISH for EBER was carried out in each sample on 5 μm-thick section as previously described (Abate et al., 2015). A control slide, prepared from a paraffin-embedded tissue block containing metastatic nasopharyngeal carcinoma in a lymph node accompanied each hybridization run.

### Laser Capture Microdissection

The neoplastic population of each sample was isolated from haematoxylin and eosin stain sections using immuno-guided laser capture microdissection to avoid the risk of including reactive lymphocytes that might alter our findings in the following analysis [i.e., quantitative reverse transcription PCR (q-PCR) and miRNA profiling]. Sections were microdissected using a PixCell IIe microscope (Arcturus Engineering, MGW, Florence, Italy) as previously described (Ambrosio et al., 2015a).

### microRNAs Profiling

Total RNA was extracted from FFPE sections of nine primary tumors using the FFPE miRNA Easy kit (Qiagen, Valencia, CA, USA), according to the manufacturer’s instructions. Small RNA libraries were prepared from 1 mg of a high-quality RNA (RNA Integrity Number ≥ 8) with the TruSeq Small RNA kit (Illumina). 1 × 36 sequencing was performed on the Illumina MiSeq platform. Thirty-six bp length raw sequences were demultiplexed using the Illumina pipeline CASAVA v1.8. A quality check of the run experiment was performed by FastQC^1^. Low quality reads and adapter sequences were trimmed off using Trimmomatic (Bolger et al., 2014). The high quality reads, with a length of 17–36 bp were clipped and subsequently aligned to the latest miRBase release (v 21 July 2014) (Kozomara and Griffiths-Jones, 2014) by Novoalign^2^. miRNA expression profiles were built by calculating the sum of read counts for each miRNA, according to the alignment criteria. Variance-stabilizing transformed count data were used to build a Euclidean distance matrix, followed by hierarchical clustering analysis to study the intra-samples correlations. miRNA differential expression analysis was performed using Bioconductor’s package DESeq (Anders and Huber, 2010). The obtained read counts for each identified miRNA were first normalized by scaling for library size factors in order to deal with variation among samples. The differential expression values were estimated using a negative binomial distribution model and local regression to estimate the relationship between the dispersion and the mean of each miRNA. Raw values were considered as statistically significant when *p*-value < 0.05. Heatmap of normalized count table and principal component analysis were performed by R. Following alignment and normalization (see above), the data was further analyzed using GeneSpring GX12 as previously described (Navari et al., 2015).

### Quantitative Reverse Transcription PCR Assay to Confirm and Quantify the Presence of Viral miRNAs

Taqman primers and probes specific for each selected viral miRNA were applied to analyse by q-PCR all cases. Total RNA was extracted using miRNAeasy FFPE kit (Qiagen, Valencia, CA, USA) according to the manufacturer’s instructions. Three T-LLs samples, currently considered not to be affected by EBV were used as negative control. RNU6B was used as endogenous control (Applied Viosystems, Applera, Italy) and the absolute expression was calculated using the 2^-Δct^ formula. Kruskal–Wallis Test was applied for statistical analysis.

### Quantitative Reverse Transcription PCR Assay to Quantify EBV Genome Load

To further strengthen our findings and prove the presence of EBV also in EBER negative samples, viral load measurement was carried out. DNA was extracted from FFPE tissue using proteinase K digestion and NucleoSpin Kit, according to the manufacturer’s instructions. Beta globin Human beta 2 microglobulin (B2m) and ApoB genes were used as a control for the efficacy of extraction and amplification of DNA from paraffin embedded tissue. According to the data from the literaturate (Junying et al., 2003) a standard curve was generated using 10-fold dilutions of Namalwa DNA varying from 500,000 to 0,5 copies of EBV DNA, assuming that Namalwa cell line is diploid and carry two copies of EBV per cell, equivalent to 3 × 10^5^ copies of EBV/μg DNA. All cases were screened by q-PCR targeting BamH1 W and EBNA-1 conserved region of EBV genome. BamH1 W targets a reiterated sequence that is present at approximately 10 copies per EBV genome and it appears to be the most sensitive method to prove the presence of viral genome, detecting low level virus. EBNA-1 targets a single copy highly conserved gene and is essential for maintaining the virus long term in dividing cells. Amplification reactions were performed in 50 μl volumes as previously described (Junying et al., 2003). Each experiment included DNA samples prepared from EBER-positive and EBER-negative cases, as well as water-only controls. Samples were considered negative if exceeded 40 cycles. The quantification results for experimental samples were extrapolated from the EBNA-1 and BAMH1 W calibration curve (Junying et al., 2003).

## Results

### Immunohistochemistry and *In situ* Hybridization Findings

**FIGURE 1:**
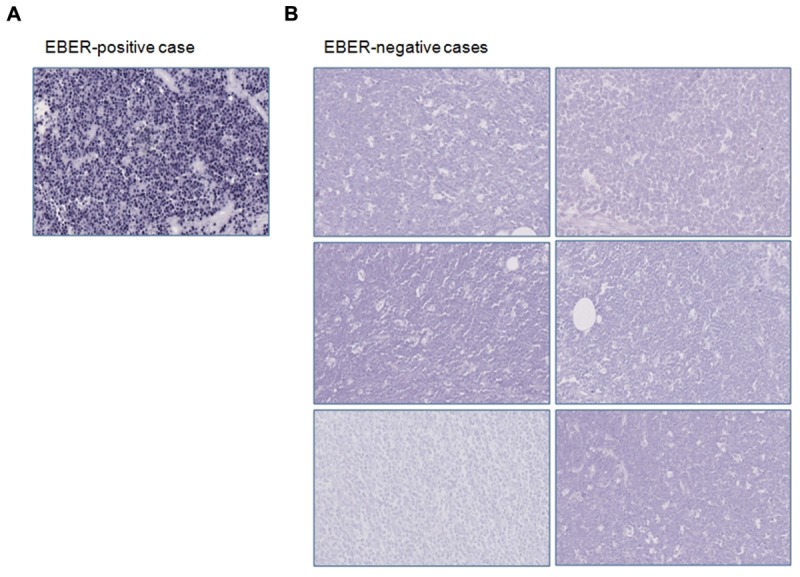
**Immunohistochemical and *in situ* hybridization findings.** **(A)** EBER-positive case is depicted; **(B)** EBER-negative specimens are shown. Original magnification: 25X.

We screened all our samples (*n* = 10) for EBV infection by performing immunohistochemistry and EBER-ISH to identify the presence of EBV-related products. Four out of 10 cases showed the typical nuclear positivity for EBNA-1 staining in almost all the neoplastic cells. By applying ISH for EBV, we observed that only four samples retained the EBV-encoded RNAs, as demonstrated by the black signal revealed in the nucleus of about 70–95% of neoplastic cells (**Figure 1A**). Six cases were negative for EBNA-1 and ISH for EBER (**Figure 1B**). In these cases no stain was detectable neither in the neoplastic cells nor in reactive small lymphocytes.

### microRNAs Expression Profiling Results and Quantitative Reverse Transcription PCR Validation

**FIGURE 2:**
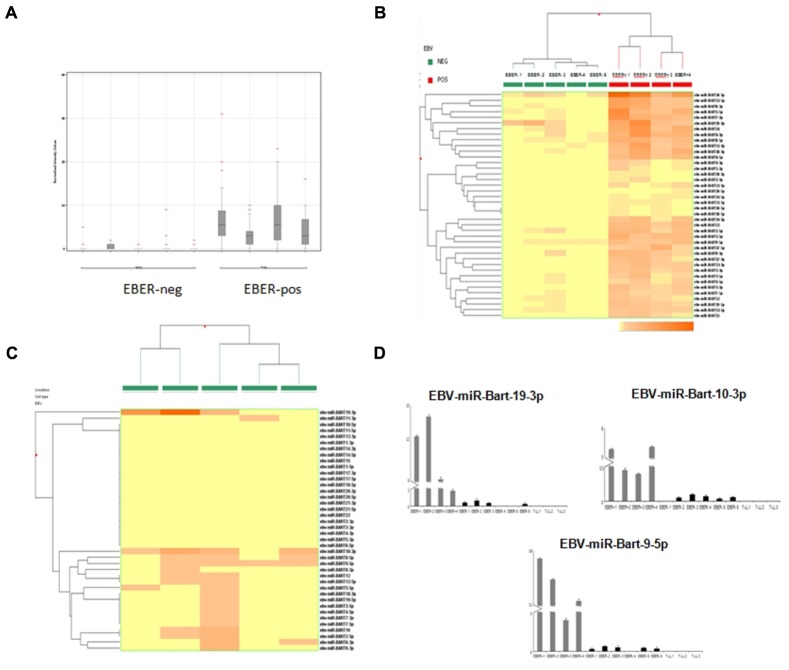
**microRNA expression profiling results and reverse transcription validation.** **(A)** Normalized intensity expression values of EBV-encoded miRNA in Burkitt lymphoma cases; expression values of all EBV-encoded miRNA are plotted for each case and are represented by boxes; bars indicate the mean values. **(B)** Unsupervised hierarchical clustering of Burkitt lymphoma cases based on the expression of EBV encoded miRNAs; the dendrogram was generated using a hierarchical clustering algorithm based on the average-linkage method. In the matrix, each column represents a sample and each row represents a miRNA. The color scale bar shows the relative miRNA expression changes normalized by the standard deviation (0 is the mean expression level of a given gene). **(C)** Unsupervised hierarchical clustering of EBER-negative Burkitt lymphoma cases based on the expression of EBV-encoded miRNAs; The dendrogram was generated using a hierarchical clustering algorithm based on the average-linkage method. In the matrix, each column represents a sample and each row represents a miRNA. The color scale bar shows the relative miRNA expression changes normalized by the standard deviation (0 is the mean expression level of a given gene). **(D)** Differential expression of EBV-encoded miRNAs in EBER-negative and EBER-positive Burkitt lymphomas *versus* control samples (lymphoblastic lymphoma) by q-PCR. Expression values are reported on the y-axis. Standard error is indicated by bars.

EBER-positive BL samples showed a clear expression of viral miRNAs but also all EBER-negative cases presented some degree of expression of at least one EBV-miRNA (**Figure 2A**). Specifically, in two cases we found four different miRNAs, in four cases we detected 2, 3, 9, and 17 miRNAs, respectively. In total, 19 different virus-encoded miRNAs were revealed. Some of them were consistently expressed in more than two samples, such as ebv-miR-BART10-3p (*n* = 4), ebv-miR-BART9-5p (*n* = 4), ebv-miR-BART19-3p (*n* = 3), ebv-miR-BART8-5p (*n* = 3). Consistently, when unsupervised hierarchical clustering of BL cases based on the expression of EBV-miRNAs was performed, EBER-negative and EBER-positive groups were clearly distinct; again, some degree of miRNA expression in EBER-negative cases was observed (**Figure 2B**). When EBER-negative BL cases were clustered alone, differences among samples were more evident with a clearly variable expression of virus encoded miRNAs (**Figure 2C**). To validate miRNA profiling results, q-PCR analysis was employed and demonstrated a significant differential expression for all the tested miRNAs, namely ebv-miR-BART9-5p (*p* = 0.006), ebv-miR-BART10-3p (*p* = 0.002), and ebv-miR-BART19-3p (*p* = 0.004), viral miRNAs being expressed in all 10 BL samples but not in T-LL specimens (**Figure 2D**).

### EBV Viral Load Measurement

**FIGURE 3:**
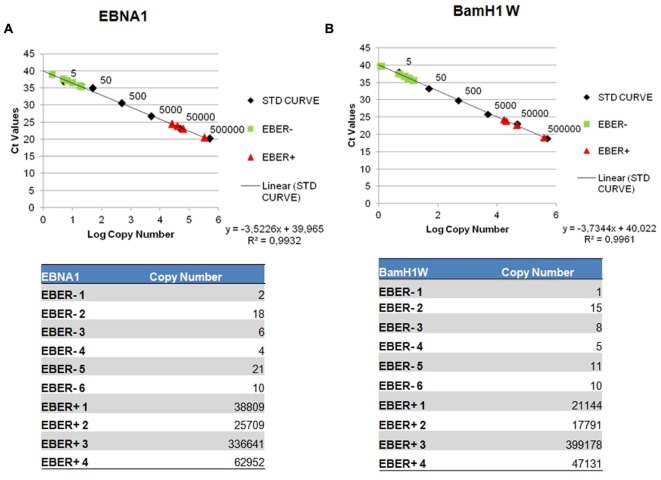
**Detection of EBV genome by reverse transcription assay.** Serial dilution of Namalwa DNA containing 500,000 to 0,5 copies of EBV genomes were amplified using primer/probe combination specific for EBV EBNA-1 **(A)** and BamH1 W conserved region **(B)**. The y-intercept corresponds to the number of cycles. The x-intercept corresponds to the copy number of each target expressed in log _10_ scale.

The quantification results were extrapolated from the EBNA-1 and BAMH1 W calibration curve where the linear relationship (*R*^2^) reached 0.998. The EBV genome was detected in 6/6 cases and all these samples contained low copy numbers of the EBV genomes, ranging of 2.07–21.03 copies/1000 cells per EBNA-1 and 1.23–15.28 copies/1000 cells per BAMH1 W (**Figures 3A,B**). Otherwise, Q-PCR analysis of EBER-positive cases revealed much higher viral loads (more than 25,000 copies/1000 cells per EBNA1 and 15,000 copies/1000 cells per BAMH1 W). EBV was regularly detectable in microdissected tumor cells from EBV-positive NPC controls while EBV was not detectable in T-LL negative controls.

## Discussion

Epstein–Barr virus is thought to play a causative role in the pathogenesis of EBV-positive BL. The etiology of cases lacking its genome in neoplastic cells is poorly understood, but is has been suggested that EBV may use a *hit-and-run* mechanism in them. EBV supplies a normal B-cell information to survive and grow that are potentially oncogenic (*hit*). A healthy immune system removes these proto-tumor cells because they express recognized viral antigens (Rochford et al., 2005). EBV can avoid elimination of infected cells by inducing epigenetic alterations and silencing of targeted viral genes in the host cell (*run*) (Queen et al., 2013; Birdwell et al., 2014). These alterations are stable and heritable and would be maintained also after the loss of the virus (Queen et al., 2013; Birdwell et al., 2014). Such ability of EBV to manipulate the host machinery to silence its own gene expression and to reshuffle the cellular epigenome resulting in long lasting cellular consequences, can act as a mechanistic framework for the *hit-and- run* oncogenesis (Queen et al., 2013; Birdwell et al., 2014). Accordingly, selection favors a proto-tumor cell that evolves to reduce its dependence on the virus from proliferation and survival by gaining complementary cellular mutations (Ambrosio et al., 2015b). In more evolved tumors, the only viral genes expressed are those poorly immunogenic and non-immunogenic (i.e., EBER and viral-encoded miRNAs). Unfortunately the standard methods to detect EBV often fail to identify also these non-immunogenic molecules, but if the virus is truly responsible for all BL, all cases should theoretically show some evidence of the infection (Queen et al., 2013). Therefore, the challenge is to devise investigative strategies to prove or exclude *hit-and-run* oncogenesis (Ambinder, 2000).

In our study we investigated this possibility by applying conventional and non-conventional tools on a series of BL cases. In fact, the main problems with the *hit-and-run* hypothesis are the absence of proof in natural setting that led to track the fate of an infected cell, and the lack of evidence in primary tumors (Stevenson et al., 2010). The only data we have concerns *in vitro* studies. It has been shown that in some cases of episome loss from BL cell lines in tissue culture, fragments of the EBV genome are incorporated into cellular DNA (Srinivas et al., 1998). Furthermore, in a series of sBL, similar fragments of the EBV genome were found in tumors that by standard criteria (EBER-ISH or EBNA-1 immunohistochemistry) would be classed as virus-negative (Srinivas et al., 1998).

The present study adds new evidence to the existing literature demonstrating that the routine methods employed to identify EBV (i.e., immunohistochemistry and EBER-ISH) are disappointing as we identified EBV genome in cases diagnosed as EBNA-1 and EBER negative (Gallagher et al., 2003; Stevenson et al., 2010). We also provide evidence to propose miRNAs detection as the most specific and sensitive tool to identify even EBV vestiges (e.g., EBV exosomes) and diagnose a previous infection in “EBV-negative” patients. According to the *hit-and-run* hypothesis, our findings support the possibility that EBV might have contributed to lymphomagenesis in our samples by initiating the oncogenic process. Then EBV genome was lost at each cell cycle and no longer detectable by conventional methods, being the tumor under the selective pressure exerted by the immune system. In fact, every population of proliferating EBV-positive cells loses 8% of the viral genomes each cell cycle; after eight cycles, only 50% of the viral DNA will remain; after 50 cycles, only 1% will persist in the population (Vereide and Sugden, 2009, 2010). The few remaining EBV genome in neoplastic cells would then be responsible for the production of the detected miRNAs. In this regard, selective release and transfer of RNA via exosomes might play a relevant role as recently demonstrated in other settings (Baglio et al., 2016). Though our findings do not represent a definitive proof of the presence of EBV inside the neoplastic cells, they highlight for the first time the possibility that EBV might contribute to the development of more cancers than simply those remaining viral genome-positive. In fact, the virus may impact on host cell homeostasis in various ways by interfering with cellular miRNAs expression and by encoding its own genes and miRNAs (Lenze et al., 2011). EBV-miRNAs may compete with miRNAs machinery and target cellular genes, thus dysregulating key pathways.

Whether confirmed on a larger cohort of cases and different tumor types, the current study may further support the rationale for strengthening the effort toward EBV vaccines that could potentially prevent the development of EBV-associated neoplasms independently of the presence or absence of viral genomes in the neoplastic cells, thus affecting the worldwide epidemiology of lymphomas. This is an idea worth considering, and seems to be realistic, because it has been demonstrated that vaccinia against EBV superficial antigens could protect from or delay EBV infection in infants (Stevenson et al., 2010).

## Author Contributions

LM analyzed data and performed the experiment, MA analyzed data and draft the work, MP carried out microRNAs profiling, GLB, LDP, and MN performed histology, extraction and quality assessment of the tissue samples. SG, MG, NO, and GDF contributed samples and tools, DG reviewed the manuscript, PP designed the study and analyzed data, LL designed the study, analyzed data and reviewed critically the manuscript. All the authors approved the final version to be published.

## Conflict of Interest Statement

The authors declare that the research was conducted in the absence of any commercial or financial relationships that could be construed as a potential conflict of interest.

## Footnotes

1 http://www.bioinformatics.babraham.ac.uk/projects/fastqc

2 http://www.novocraft.com
